# Supplementary material for: The impact of social ties and SARS memory on the public awareness of 2019 novel coronavirus (SARS-CoV-2) outbreak
Source: Sci Rep. 2020 Oct 26;10:18241. doi: 10.1038/s41598-020-75318-9 (PMC7589561; doi:10.1038/s41598-020-75318-9)
Supplement: Supplementary file 1 — Supplementary Information 1. [file 41598_2020_75318_MOESM1_ESM.docx]

**Supplementary Materials**:

File S1: Baidu Search Index data.

File S2: The data necessary to replicate the analysis of this study.

File S3: The model selection using AIC.
