# Supplementary material for: The impact of social ties and SARS memory on the public awareness of 2019 novel coronavirus (SARS-CoV-2) outbreak
Source: Sci Rep. 2020 Oct 26;10:18241. doi: 10.1038/s41598-020-75318-9 (PMC7589561; doi:10.1038/s41598-020-75318-9)
Supplement: Supplementary file 4 — Supplementary Information 4. [file 41598_2020_75318_MOESM4_ESM.docx]

**The impact of social ties and SARS memory on the public awareness of 2019 novel coronavirus (SARS-CoV-2) outbreak**

Haohui “Caron” Chen^1,2*^, Cecile Paris^1^, Andrew Reeson^1^

**Affiliations:**

^1^Data61, Commonwealth Scientific and Industrial Research Organization, Australia

^2^Monash University, Australia

*Correspondence to: Haohui Chen (Caronhaohui.chen@data61.csiro.au).

# Eq.4 Model selection using AIC

## Using stepAIC() from ‘MASS’ package in R

Start: AIC=1026.61

$$\Delta t_{i}= {GDP\_per\_capita}_{i}+{SubProvincial}_{i}+ D_{i}+d_{i}+{SARS}_{i}+D_{i}* {SARS}_{i}+d_{i}* {SARS}_{i}$$

|  |  | Df | Sum of Sq | RSS | AIC |
| --- | --- | --- | --- | --- | --- |
| - | $d_{i}* {SARS}_{i}$ | 1 | 26.74 | 8408.2 | 1025.6 |
|  | <none> |  |  | 8381.5 | 1026.6 |
| - | $D_{i}* {SARS}_{i}$ | 1 | 130.4 | 8511.9 | 1029.3 |
| - | ${GDP\_per\_capita}_{i}$ | 1 | 593.34 | 8974.8 | 1045.5 |
| - | ${SubProvincial}_{i}$ | 1 | 1601.37 | 9982.8 | 1077.9 |

Step: AIC=1025.58

$$\Delta t_{i}= {GDP\_per\_capita}_{i}+{SubProvincial}_{i}+ D_{i}+d_{i}+{SARS}_{i}+D_{i}* {SARS}_{i}$$

|  |  | Df | Sum of Sq | RSS | AIC |
| --- | --- | --- | --- | --- | --- |
|  | <none> |  |  | 8408.2 | 1025.6 |
| - | $D_{i}* {SARS}_{i}$ | 1 | 112.44 | 8520.7 | 1027.6 |
| - | $d_{i}$ | 1 | 356.69 | 8764.9 | 1036.2 |
| - | ${GDP\_per\_capita}_{i}$ | 1 | 623.8 | 9032 | 1045.4 |
| - | ${SubProvincial}_{i}$ | 1 | 1587.93 | 9996.1 | 1076.3 |

## Using aictab() from ‘AICcmodavg’ package in R

Model selection based on AICc:

|  | K | AICc | Delta_AICc | AICcWt | Cum.Wt | LL |
| --- | --- | --- | --- | --- | --- | --- |
| fit6 | 8 | 1893.62 | 0 | 0.64 | 0.64 | -938.57 |
| fit7 | 9 | 1894.77 | 1.15 | 0.36 | 1 | -938.08 |
| fit5 | 7 | 1990.93 | 97.31 | 0 | 1 | -988.28 |
| fit3 | 5 | 1992.24 | 98.63 | 0 | 1 | -991.02 |
| fit2 | 3 | 2006.2 | 112.58 | 0 | 1 | -1000.06 |
| fit4 | 3 | 2080.25 | 186.63 | 0 | 1 | -1037.09 |
| fit1 | 3 | 2093.7 | 200.08 | 0 | 1 | -1043.81 |

# Eq.5 Model selection using AIC

## Using stepAIC() from ‘MASS’ package in R

Start: AIC=444.43

$$O_{t_{warning(i)}}^{COVID-19}= {GDP\_per\_capita}_{i}+{SubProvincial}_{i}+ D_{i}+ d_{i}+{SARS}_{i}+D_{i}* {SARS}_{i}+d_{i}* {SARS}_{i}$$

|  |  | Df | Sum of Sq | RSS | AIC |
| --- | --- | --- | --- | --- | --- |
|  | <none> |  |  | 1202.5 | 444.43 |
| - | $D_{i}* {SARS}_{i}$ | 1 | 31.09 | 1233.6 | 451 |
| - | ${GDP\_per\_capita}_{i}$ | 1 | 79.68 | 1282.2 | 463.99 |
| - | ${SubProvincial}_{i}$ | 1 | 126.34 | 1328.9 | 475.99 |
| - | ${d_{i}*SARS}_{i}$ | 1 | 904.56 | 2107.1 | 630.88 |

## Using aictab() from ‘AICcmodavg’ package in R

Model selection based on AICc:

|  | K | AICc | Delta_AICc | AICcWt | Cum.Wt | LL |
| --- | --- | --- | --- | --- | --- | --- |
| fit6 | 9 | 1400.51 | 0 | 1 | 1 | -690.98 |
| fit5 | 7 | 1475.5 | 74.99 | 0 | 1 | -730.58 |
| fit4 | 3 | 1697.07 | 296.56 | 0 | 1 | -845.5 |
| fit3 | 5 | 1777.33 | 376.82 | 0 | 1 | -883.57 |
| fit2 | 3 | 1787.04 | 386.53 | 0 | 1 | -890.48 |
| fit1 | 3 | 1813.52 | 413.01 | 0 | 1 | -903.72 |

# Eq.6 Model selection using AIC

## Using stepAIC() from ‘MASS’ package in R

Start: AIC= -1166.37

$${\Delta O}_{i}= {GDP\_per\_capita}_{i}+{SubProvincial}_{i}+ D_{i}+d_{i}+ {SARS}_{i}+D_{i}* {SARS}_{i}+d_{i}* {SARS}_{i}$$

|  |  | Df | Sum of Sq | RSS | AIC |
| --- | --- | --- | --- | --- | --- |
| - | $d_{i}* {SARS}_{i}$ | 1 | 0.00632 | 9.6965 | -1168.2 |
|  | <none> |  |  | 9.6902 | -1166.4 |
| - | $D_{i}* {SARS}_{i}$ | 1 | 0.24535 | 9.9355 | -1160 |
| - | ${GDP\_per\_capita}_{i}$ | 1 | 0.30485 | 9.995 | -1158 |
| - | ${SubProvincial}_{i}$ | 1 | 0.77211 | 10.4623 | -1142.8 |

Step: AIC=-1168.15

$${\Delta O}_{i}= {GDP\_per\_capita}_{i}+{SubProvincial}_{i}+ D_{i}+d_{i}+ {SARS}_{i}+D_{i}* {SARS}_{i}$$

|  |  | Df | Sum of Sq | RSS | AIC |
| --- | --- | --- | --- | --- | --- |
|  | <none> |  |  | 9.6965 | -1168.2 |
| - | $d_{i}$ | 1 | 0.05967 | 9.7562 | -1168.1 |
| - | $D_{i}* {SARS}_{i}$ | 1 | 0.27238 | 9.9689 | -1160.9 |
| - | ${GDP\_per\_capita}_{i}$ | 1 | 0.31556 | 10.012 | -1159.5 |
| - | ${SubProvincial}_{i}$ | 1 | 0.83867 | 10.5352 | -1142.5 |

## Using aictab() from ‘AICcmodavg’ package in R

Model selection based on AICc:

|  | K | AICc | Delta_AICc | AICcWt | Cum.Wt | LL |
| --- | --- | --- | --- | --- | --- | --- |
| fit6 | 9 | -215.96 | 0 | 1 | 1 | 117.26 |
| fit3 | 5 | -190.92 | 25.04 | 0 | 1 | 100.55 |
| fit5 | 7 | -190.14 | 25.83 | 0 | 1 | 102.24 |
| fit2 | 3 | -184.96 | 31 | 0 | 1 | 95.52 |
| fit4 | 3 | -182.83 | 33.13 | 0 | 1 | 94.45 |
| fit1 | 3 | -182.53 | 33.44 | 0 | 1 | 94.3 |
